# Quantitation of Productively Infected Monocytes and Macrophages of Simian Immunodeficiency Virus-Infected Macaques

**DOI:** 10.1128/JVI.00290-16

**Published:** 2016-05-27

**Authors:** Claudia R. Avalos, Sarah L. Price, Ellen R. Forsyth, Julia N. Pin, Erin N. Shirk, Brandon T. Bullock, Suzanne E. Queen, Ming Li, Dane Gellerup, Shelby L. O'Connor, M. Christine Zink, Joseph L. Mankowski, Lucio Gama, Janice E. Clements

**Affiliations:** a Department of Molecular and Comparative Pathobiology, Johns Hopkins University School of Medicine, Baltimore, Maryland, USA; b Department of Pathology, Johns Hopkins University School of Medicine, Baltimore, Maryland, USA; c Department of Neurology, Johns Hopkins University School of Medicine, Baltimore, Maryland, USA; d Department of Pathology and Laboratory Medicine, University of Wisconsin School of Medicine, Madison, Wisconsin, USA; Emory University School of Medicine

## Abstract

Despite the success of combined antiretroviral therapy (ART), human immunodeficiency virus (HIV) infection remains a lifelong infection because of latent viral reservoirs in infected patients. The contribution of CD4^+^ T cells to infection and disease progression has been extensively studied. However, during early HIV infection, macrophages in brain and other tissues are infected and contribute to tissue-specific diseases, such as encephalitis and dementia in brain and pneumonia in lung. The extent of infection of monocytes and macrophages has not been rigorously assessed with assays comparable to those used to study infection of CD4^+^ T cells and to evaluate the number of CD4^+^ T cells that harbor infectious viral genomes. To assess the contribution of productively infected monocytes and macrophages to HIV- and simian immunodeficiency virus (SIV)-infected cells *in vivo*, we developed a quantitative virus outgrowth assay (QVOA) based on similar assays used to quantitate CD4^+^ T cell latent reservoirs in HIV- and SIV-infected individuals in whom the infection is suppressed by ART. Myeloid cells expressing CD11b were serially diluted and cocultured with susceptible cells to amplify virus. T cell receptor β RNA was measured as a control to assess the potential contribution of CD4^+^ T cells in the assay. Virus production in the supernatant was quantitated by quantitative reverse transcription-PCR. Productively infected myeloid cells were detected in blood, bronchoalveolar lavage fluid, lungs, spleen, and brain, demonstrating that these cells persist throughout SIV infection and have the potential to contribute to the viral reservoir during ART.

**IMPORTANCE** Infection of CD4^+^ T cells and their role as latent reservoirs have been rigorously assessed; however, the frequency of productively infected monocytes and macrophages *in vivo* has not been similarly studied. Myeloid cells, unlike lymphocytes, are resistant to the cytopathic effects of HIV. Moreover, tissue-resident macrophages have the ability to self-renew and persist in the body for months to years. Thus, tissue macrophages, once infected, have the characteristics of a potentially stable viral reservoir. A better understanding of the number of productively infected macrophages is crucial to further evaluate the role of infected myeloid cells as a potential viral reservoir. In the study described here we compared the frequency of productively infected CD4^+^ T cells and macrophages in an SIV-infected macaque model. We developed a critical assay that will allow us to quantitate myeloid cells containing viral genomes that lead to productive infection in SIV-infected macaques and assess the role of macrophages as potential reservoirs.

## INTRODUCTION

Lentiviruses infect myeloid lineage cells in tissues, and these cells are responsible for the multiorgan disease characteristic of infection with this family of retroviruses (1–3). Human immunodeficiency virus (HIV) was the first primate lentivirus identified that infects CD4^+^ T cells as well as myeloid cells in the blood and tissues of infected individuals (4–6). HIV infects myeloid cells in lymph nodes, spleen, heart, lungs, the peripheral nervous system, and the central nervous system (CNS) (7–11). The HIV genome encodes genes that specifically interact and/or interfere with restriction factors present in myeloid cells, providing evolutionary evidence that HIV replication in myeloid cells is important for virus replication and pathogenesis *in vivo* (12).

Myeloid cells were thought to be terminally differentiated cells with a limited life span. However, recent studies have demonstrated that resident tissue macrophages are capable of self-renewal and that monocytes from blood differentiate into distinct macrophage phenotypes after entering tissues (13, 14). Moreover, tissue-resident macrophages, such as alveolar macrophages, splenic red pulp macrophages, and microglia, are derived from embryonic yolk sac progenitor cells that self-renew with little to no contribution from circulating monocytes during homeostasis (15–18). Furthermore, HIV- and simian immunodeficiency virus (SIV)-infected macrophages are not efficiently killed by CD8^+^ T cells like infected CD4^+^ T cells are (19, 20). Thus, resident tissue macrophages remain in tissues long term, are relatively resistant to the cytopathic effects of HIV infection compared to CD4^+^ T cells, and may serve as stable viral reservoirs.

SIV-infected macaques have been used to study the pathogenesis of SIV *in vivo* and have been used as models of HIV infection in humans. Like HIV, SIV infects both CD4^+^ T cells and macrophages in blood, tissues, and brain (21–25). Our laboratories developed and characterized a consistent, accelerated SIV-infected macaque model resulting in AIDS and CNS disease (in ∼80% of macaques) in 3 months, which is shorter than the course of disease pathogenesis and frequency of CNS disease in other models of SIV infection (21). Another model used to study CNS infection used depletion of CD8^+^ T cells in SIV-infected macaques, which resulted in the increased accumulation of infected macrophages in the CNS and a heightened severity of neurological disease, suggesting that infection of macrophages plays a key role in CNS disease (26).

The frequency of HIV or SIV infection of macrophages in tissues has previously been examined in a number of studies (27, 28). Infection is quantified by measuring the amount of viral DNA in cells isolated from tissues; however, this approach overestimates the number of productively infected CD4^+^ T cells due to the presence of a large proportion of defective proviruses *in vivo* (29, 30). A more rigorous approach to the quantification of cells that harbor replication-competent virus is the quantitative viral outgrowth assay (QVOA), which quantitates the number of HIV-infected resting CD4^+^ T cells that produce infectious virus (31–33). This assay has been used to quantify the number of resting CD4^+^ lymphocytes *in vivo* in HIV-infected individuals on antiretroviral therapy (ART) that harbor replication-competent viral genomes and serves as one of the major assays for studying viral latency in that cell type (29, 31).

Using a QVOA that our laboratories developed for SIV-infected nonhuman primate CD4^+^ T cells, we previously demonstrated that the number of infected resting CD4^+^ T cells in the blood and tissues of macaques in which SIV infection was suppressed by ART was equivalent to the number of infected resting CD4^+^ T cells in HIV-infected humans on ART (31–35). In this study, we developed a novel macrophage QVOA (Mϕ-QVOA) to assess the frequency of productively SIV-infected monocytes (in blood) and macrophages (in tissues) using a well-characterized SIV-infected macaque model (36–38). To determine the potential contribution of CD4^+^ T cells to the quantitation of macrophages, we also assessed the number of CD3^+^ T cells in each assay by measuring the amount of T cell receptor β (TCRβ) RNA.

Using the myeloid Mϕ-QVOA, we show that during chronic SIV infection, productively infected monocytes or macrophages are present in blood, bronchoalveolar lavage (BAL) fluid, lungs, spleen, and brain. This assay can also be used to quantify myeloid cells productively infected with HIV and SIV that may contribute to viral persistence and latency in ART-treated humans and macaques.

## MATERIALS AND METHODS

### Animal studies.

Fourteen juvenile pigtailed macaques (Pms; Macaca nemestrina) were inoculated intravenously with the SIV/DeltaB670 swarm and the macrophage-tropic clone SIV/17E-Fr as previously described (36, 38, 39). One macaque (Pm11) was treated at 12 days postinoculation (dpi) with 12.5 mg fluconazole and 5 mg paroxetine once per day orally and euthanized at ∼80 dpi as previously described (40). Three macaques (Pm12, Pm13, and Pm14) were treated at 28 days prior to virus inoculation with 2 mg minocycline/kg of body weight twice per day orally and were euthanized during asymptomatic infection (∼35 dpi). These macaques were added to the study to investigate macrophage infection at different times after infection. These treatments did not affect either the plasma viral load or disease progression (40). Eleven macaques were euthanized during late-stage infection (50 to 87 dpi), and three macaques were euthanized during chronic infection (34 to 36 dpi) (Table 1). Blood and cerebrospinal fluid (CSF) samples were collected longitudinally postinfection. When macaques were euthanized, they were perfused with sterile saline to remove blood and circulating virus as described elsewhere (35). Viral loads in plasma and CSF, CD4^+^ T cell counts in blood, and viral RNA levels in tissues were determined for all macaques in the study (Table 1). To assess the extent of CNS inflammation and pathology, brain tissue was evaluated as previously described and the degree of inflammation was scored on a scale ranging from none to severe (38). These studies were performed in accordance with federal guidelines and institutional policies and approved by the Johns Hopkins School of Medicine Animal Care and Use Committee.

**TABLE 1:** Detailed characterization of the SIV-infected macaques used in the study^*c*^

Animal identifier	Duration of infection (days)	CNS score	Cell count (no. of cells/μl blood)		Viral load(no. of SIV copies/ml) in:		Tissue viral load (no. of SIV copies/μg tissue RNA)			
			CD4^+^ T cells	Monocytes	Plasma	CSF	Parietal cortex	Basal ganglion	Lung	Spleen
Pm1	62	Severe	166	1,271	6.50E+07	9.85E+06	5.65E+06	4.10E+06	74,024	2.59E+06
Pm2	60	Severe	57	45	8.83E+07	2.43E+07	1.30E+06	1.11E+06	99,416	675,000
Pm3	50	Severe	113	1,022	4.24E+07	4.98E+06	826,254	1.55E+06	503,785	1.22E+06
Pm4	84	Severe	52	4,333	1.49E+09	1.14E+07	4.63E+06	1.92E+06	1,543	1.61E+07
Pm5	86	Mild	222	493	1.18E+07	1.84E+07	1.48E+06	807,774	247	1.19E+07
Pm6	85	None	396	525	3.65E+08	166,022	699	10,632	1,197	8.45E+06
Pm7	83	None	418	767	1.05E+07	117,088	22	14,995	332	8.68E+06
Pm8	79	None	56	416	2.20E+06	129,536	ND	ND	129	5.51E+06
Pm9	87	None	464	874	1.25E+06	112,867	ND	ND	152	4.40E+06
Pm10	84	None	693	1,071	300,229	12,105	ND	ND	1,797	204,000
Pm11^*a*^	84	None	119	612	651,191	142,755	196	ND	282	268,000
Pm12^*b*^	34	None	681	481	4.55E+08	20,761	ND	ND	741	866,000
Pm13^*b*^	35	None	390	350	1.72E+08	8.41E+07	3,365	96,705	421	1.62E+06
Pm14^*b*^	36	None	758	588	8.37E+07	1.33E+06	1,364	586	253	377,000

a The animal was treated with fluconazole and paroxetine at day 12 postinoculation.

b The animal was pretreated with minocycline at 28 days prior to infection.

c The treatments did not affect virus replication or progression of disease. Abbreviations: ND, not detected (the level was below the limit of detection); Pm, pigtailed macaque.

### Isolation of myeloid cells and lymphocytes from blood and tissues.

Peripheral blood mononuclear cells (PBMCs) were isolated by density gradient centrifugation on a 1.077-g/ml Percoll/Hanks gradient (GE Healthcare, Pittsburgh, PA) according to the manufacturer's protocol. BAL fluid was obtained by passing 250 ml of sterile saline (Life Technologies, Grand Island, NY) into the lungs via a bronchoscope. BAL fluid cells were isolated by passing the lavage samples through a 183-μm-pore-size sterile mesh. Spleen and lung cells were mechanically removed from tissues using an 18-gauge needle and passed through a 100-μm-mesh-size cell strainer. Brain parenchymal macrophages and microglia were isolated as previously described (41).

Blood, BAL fluid, lung, and spleen macrophages were cultured in RPMI 1640 medium (Life Technologies) supplemented with 20% heat-inactivated human type AB serum (Gemini Bio Products, West Sacramento, CA), 100 U/ml penicillin-streptomycin (Life Technologies), 20 μg/ml gentamicin (Life Technologies), 2 mM l-glutamine (Life Technologies), 2 mM sodium pyruvate (Sigma), 10 mM HEPES buffer (Life Technologies), and 50 ng/ml recombinant human macrophage colony-stimulating factor (M-CSF; R&D, Minneapolis, MN). Brain macrophages were cultured in Dulbecco modified Eagle medium (Life Technologies) supplemented with 5% heat-inactivated fetal bovine serum (FBS; Atlanta Biologicals), 5% IS giant cell tumor conditioned medium (Irvine Scientific, Santa Ana, CA), 100 U/ml penicillin-streptomycin (Life Technologies), 70 μg/ml gentamicin (Life Technologies), 2 mM l-glutamine (Life Technologies), 3 mM sodium pyruvate (Sigma), and 10 mM HEPES buffer (Life Technologies).

CD4^+^ T cells were cultured in RPMI 1640 medium supplemented with 10% heat-inactivated bovine serum (Atlanta Biologicals), 100 U/ml penicillin-streptomycin (Life Technologies), 1% T cell growth factor (31), and 100 U/ml interleukin-2 (IL-2; Novartis, New York, NY). Samples were analyzed fresh or frozen viably and rapidly thawed in the corresponding medium prior to cell isolation.

### Mϕ-QVOA.

Monocytes and tissue macrophages strongly express the integrin CD11b (42), a common myeloid marker (43–46). Myeloid cells were purified on the basis of expression of CD11b with a nonhuman primate CD11b antibody-conjugated microbead kit (Miltenyi Biotec, Auburn, CA) according to the manufacturer's protocol. The purified macrophages were cultured in triplicate in a 10-fold limiting dilution in the presence of 10 μM zidovudine (Sigma) and 25 nM darunavir (Janssen, Titusville, NJ) for 3 days for cell attachment or 7 days for differentiation of peripheral blood monocytes. Poly-l-lysine-coated plates (Sigma) were used, and the plates were spun down at 2,000 rpm (872 × *g*) for 10 min to increase cell adherence. The cells were washed twice with Hanks balanced salt solution (Life Technologies) to remove nonadherent CD3^+^ lymphocytes. Medium containing 10 ng/ml recombinant human tumor necrosis factor alpha (TNF-α; ProSpec, East Brunswick, NJ) and 1 × 10^5^ CEMx174 cells/well was added to each well. CEMx174 cells served to expand the virus released from infected cells, as previously described (47). The medium was replenished with TNF-α after 4 days in coculture; supernatants and cell lysates were collected following 12 days of coculture with CEMx174 cells. The presence of replication-competent virus was determined by isolating RNA from the supernatant and measuring the amount of SIV RNA by quantitative reverse transcription-PCR (qRT-PCR). The frequency of cells harboring replication-competent virus was determined by the use of limiting dilution assay statistics (32) and expressed as the number of infectious units per million (IUPM). The contents of two sets of negative-control wells with CEMx174 cells only were added to the cultures. In addition, to determine the number of CD3^+^ T cells that were in the assay, the contents of duplicate control wells with CD11b^+^ macrophages without CEMx174 cells were used to measure the amount of TCRβ RNA, as described below.

### Generation of RNA standards for TCRβ RNA assay.

RNA isolated from macaque CD3^+^ T cells was reverse transcribed into cDNA using a SuperScript II enzyme kit (Life Technologies) with 4.1 mM MgCl_2_, 0.5 mM (each) deoxynucleoside triphosphate, 1 mM dithiothreitol, 150 ng random hexamers, 1× first-strand buffer (provided in kit), and 1 unit of RNaseOUT in a 20-μl reaction mixture. The sample was incubated at 25°C for 15 min, 42°C for 40 min, 85°C for 10 min, and 25°C for 10 min. The resulting cDNA was amplified by PCR using a PCR SuperMix high-fidelity kit (Life Technologies) with TCRβ-specific primers, forward primer 5′-GAG GAC CTG AAA AAG GTG TTC-3′ and reverse primer 5′-CAT AGA GGA TGG TGG CAG ACA-3′, designed to be specific for the constant region of the TCRβ chain of macaques, as previously described (48). The mix was incubated for 30 s at 94°C, followed by 35 cycles of 94°C for 15 s, 50°C for 15 s, and 68°C for 30 s, and was then incubated at 72°C for 10 min. The resulting TCRβ PCR product was cloned into a pCR2.1 TOPO vector and sequenced, and the sequence was confirmed. For *in vitro* RNA transcription, the plasmid was digested with BamHI, and TCRβ RNA was then generated with a MEGAscript T7 kit (Life Technologies) and used as the control transcript for preparation of the standard curve.

### Quantitation of TCRβ RNA.

TCRβ RNA was quantitated by qRT-PCR using a QuantiTect kit (Qiagen), the above-described primers, and the probe 5′-/56-FAM/ACT TCC GCT/ZEN/GCC AAG TCC AGT TCT AT/3IABkFQ/-3′ (where 56-FAM is 6-carboxyfluorescein, 3IABkFQ is 3′ Iowa Black FQ, and Zen is an internal quencher [Integrated DNA Technologies, Coralville, Iowa]), based on sequence analyses of macaque TCRβ RNA. Cycling conditions were as follows: 50°C for 30 min, 95°C for 15 min, and 45 cycles of 94°C for 15 s, 55°C for 15 s, and 60°C for 30 s. 18S rRNA was multiplexed with the TCRβ RNA to control for cell counts. To determine the average number of TCRβ copies per CD3^+^ T cell, PBMCs from seven uninfected macaques were labeled with phycoerythrin (PE)-conjugated anti-CD3 clone SP34 (BD Bioscience, San Jose, CA) and magnetically separated using an EasySep PE positive selection kit (Stemcell Technologies, Vancouver, BC, Canada). The purity of the cells was confirmed by flow cytometry (see Fig. 2A). A minimum of 4 aliquots of a million CD3^+^ T cells from each macaque was used to isolate RNA that was analyzed by qRT-PCR for TCRβ RNA, and the number of copies of TCRβ RNA per macaque CD3^+^ T cell was calculated (see Fig. 2B).

### Flow cytometry.

All CD11b^+^ myeloid cells were labeled with PE-conjugated anti-CD3 clone SP34 (BD Bioscience) and fluorescein isothiocyanate-conjugated anti-CD11b clone Bear1 (Beckman Coulter, Brea, CA) to assess the selection efficiency. Purified CD4^+^ T cells were stained with antibodies for HLA-DR clone L243 (BioLegend), CD3 clone SP34-2 (BD Bioscience), CD4 clone OKT4 (BioLegend), CD8 clone RPA-T8 (BioLegend), and TCRγδ clone B1.1 (eBioscience). Cells were stained for 20 min at room temperature in 100 μl phosphate-buffered saline–2% FBS and fixed for 10 min with Fix/Lyse buffer (Becton Dickinson, Franklin Lakes, NJ). After fixation, samples were analyzed in a BD LSRFortessa flow cytometer using DIVA software (Becton Dickinson, Franklin Lakes, NJ). The gating of CD3^+^ T cells was easily visualized as small CD3^+^ nonautofluorescent cells. All data were analyzed using FlowJo software. CD4^+^ T cell and monocyte counts were analyzed as previously described (38).

### Fluorescence microscopy.

Cocultured live monocyte-derived macrophages were treated at 37°C for 4 h with 10 μM pHrodo Green Escherichia coli bioparticles (Life Technologies), which can be phagocytosed only by functional macrophages and are nonfluorescent at neutral pH but which are fluorescent in the acidic pH of phagosomes (49). The cells were then stained at room temperature for 20 min with 2 drops/ml NucBlue live nuclear marker (Life Technologies), a Hoechst 33342 nuclear marker that emits fluorescence when bound to DNA (50). Images were taken on a Nikon Eclipse TE200 fluorescence microscope and merged using Adobe Photoshop CS4 software (Adobe, San Jose, CA).

### T cell viral outgrowth assay.

Total CD4^+^ T cells were enriched by use of a nonhuman primate-specific microbead isolation kit (Miltenyi Biotec), which depleted cells expressing CD8, CD11b, CD16, CD20, CD56, and CD66abce. Infected CD4^+^ T cells were quantified by using a previously described 5-fold limiting dilution assay (34, 35, 47). The cells were cocultured with CEMx174 cells for 2 weeks. The culture supernatant was analyzed for SIV RNA by qRT-PCR. The frequencies of infected cells were determined by limiting dilution assay statistics (32) and were expressed in terms of the number of IUPM.

### RNA isolation from cells and tissues.

RNA was isolated from cell cultures with an RNeasy Plus minikit (Qiagen, Valencia, CA) according to the manufacturer's protocol, with modifications. An on-column DNase digestion was performed using an RNase-free DNase kit (Qiagen) with the addition of 4 units of Turbo DNase (Life Technologies) to the enzyme mix. Two hundred microliters of fluid (from CSF, plasma, and culture supernatants) was isolated using a QIAamp MinElute virus spin kit (Qiagen) according to the manufacturer's protocol, with modifications. An on-column DNase digestion was performed using the RNase-free DNase kit (Qiagen) with the addition of 3 units of RQ1 DNase (Promega, Madison, WI) to the enzyme mix.

Frozen tissues were isolated with RNase STAT-60 (Tel Test Inc., Friendswood, TX) and homogenized with a FastPrep-24 instrument (MP Biomedicals, Santa Ana, CA) in lysing matrix D tubes (MP Biomedicals). The sample was separated with chloroform, and the aqueous phase was treated with isopropanol to precipitate the RNA. The RNA was purified with an RNeasy minikit (Qiagen) with an on-column DNase digestion using the RNase-free DNase kit (Qiagen) and the addition of 3 units of RQ1 DNase (Promega) to the enzyme mix.

### Quantitation of SIV RNA.

SIV RNA was measured by qRT-PCR using a QuantiTect virus kit (Qiagen) and primers specific for the SIV *gag* region, as previously described (34, 51, 52). Three reactions were performed for each sample. To control for DNA contamination, one reaction was analyzed by use of a reaction mixture without reverse transcriptase. Samples were analyzed using a Rotor-Gene thermocycler (Qiagen).

### PBMC infection.

PBMCs from uninfected pigtailed macaques were isolated by use of a Percoll density gradient and plated in 48-well plates in RPMI 1640 medium supplemented with 2 μg/ml recombinant human IL-2 (Life Technologies) and 2 μg/ml phytohemagglutinin, M form (Life Technologies), overnight. The PBMCs were spinoculated for 2 h with 100 μl supernatants from triplicate independent wells from Mϕ-QVOAs with samples from Pm6 and Pm4 (blood, spleen, microglia, and lung). The PBMCs were infected for 5 h at 37°C, the supernatant was removed, and excess virus was washed five times with sterile saline. The medium was replaced and supplemented with 2 μg/ml IL-2 (Life Technologies). Supernatants were collected at days 5, 10, and 13 postinoculation.

### SIV *env* sequence analyses.

Supernatant RNA was reverse transcribed into cDNA using a SuperScript III reverse transcriptase enzyme kit (Life Technologies) according to the manufacturer's protocol. The resulting cDNA was amplified using a Platinum PCR SuperMix high-fidelity kit (Life Technologies), according to the manufacturer's protocol, with two rounds of nested PCR against SIV *env*-specific primers. The primer sequences were as follows: for the 1st, round 5′-ARG AAT GCG ACA ATT CCC CT-3′ for the forward primer and 5′-TCC ATC ATY CTT GTG CAT GAA G-3′ for the reverse primer, and for the 2nd round, 5′-CAG TCA CAG AAC AGG CAA TAG A-3′ for the forward primer and 5′-TAA GCA AAG CAT AAC CTG GMG GT-3′ for the reverse primer. In both rounds, amplification was with the same cycling conditions: 94°C for 1 min and then 40 cycles of 94°C for 30 s, 55°C for 30 s, and 72°C for 1 min. The resulting product was ∼560 bp. The PCR product was purified using a DNA Clean & Concentrator-5 kit (Zymo Research) according to the manufacturer's protocol and sequenced on an Illumina MiSeq sequencer. The sequences were analyzed using Geneious (version 8.0) software (Biomatters, Auckland, New Zealand).

### Statistics.

The frequencies of infected cells in limiting dilution assays were calculated using the IUPMStats (version 1.0) infection frequency calculator (http://silicianolab.johnshopkins.edu) (32). Correlations were computed using a two-tailed nonparametric Spearman rank correlation analysis. Statistical analyses were performed using Prism software (GraphPad Software, La Jolla, CA).

## RESULTS

### Development of Mϕ-QVOAs.

The viral outgrowth assay used to measure productively infected HIV- and SIV-infected resting CD4^+^ T cells uses highly purified resting CD4^+^ T cells that are serially diluted and activated with IL-2 (31, 33). To amplify infectious virus produced by CD4^+^ T cells, susceptible cells of human cell lines (MT4 or CEMx174 cells) are added to each well and the virus in the cell supernatants is quantified by qRT-PCR or enzyme-linked immunosorbent assay (33–35, 47). The frequency of productively infected cells is calculated on the basis of the number of virus- or viral RNA-positive wells or replicates in each of the serial dilutions (32, 33).

**FIG 1:**
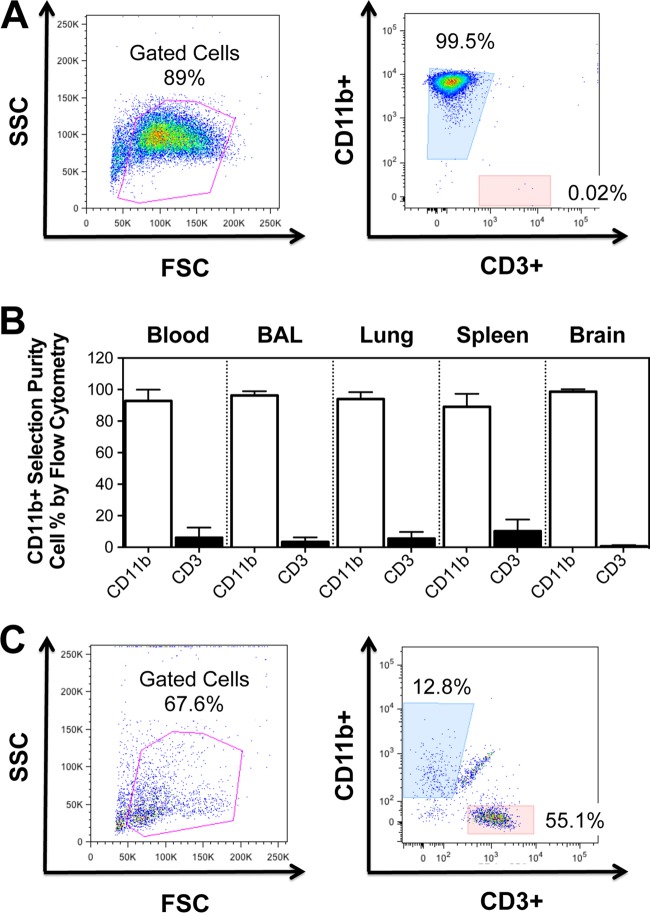
Analysis of CD11b^+^ monocytes and macrophages. Cells expressing CD11b were stained with antibodies to CD11b and CD3 to identify myeloid cells and lymphocytes. (A) Representative flow cytometry dot plots of the cell population after isolation with nonhuman primate-specific beads (left) and the highly purified population of CD11b^+^ monocytes (right). (B) The frequency of CD11b^+^ cells and CD3^+^ cells in the indicated compartment was determined by flow cytometry. (C) Nonadherent cells from Mϕ-QVOAs were removed and analyzed by flow cytometry. Flow cytometry dot plots are shown for a representative animal. Cells were analyzed by forward scatter (FSC) and side scatter (SSC) (left) and for macrophage and T cell markers (right). Data are presented as means with standard deviations.

Our macrophage quantitative viral outgrowth assay (Mϕ-QVOA) was based on the same experimental approach used for the CD4^+^ T cell QVOA. Monocytes and tissue macrophages strongly express the integrin CD11b (42) and could be separated from other cell types by sorting with CD11b-specific Miltenyi magnetic beads (43–46). The CD11b^+^ cells were serially diluted, and antiretroviral drugs were added to the culture to prevent virus spread from nonadherent CD4^+^ T cells. Unlike T cells, macrophages do not divide exponentially when activated in culture and require adherence to culture plates when grown *in vitro* (53). After the monocytes or macrophages adhered to the culture plate, residual nonadherent T cells were removed from each well (Fig. 1C).

**FIG 2:**
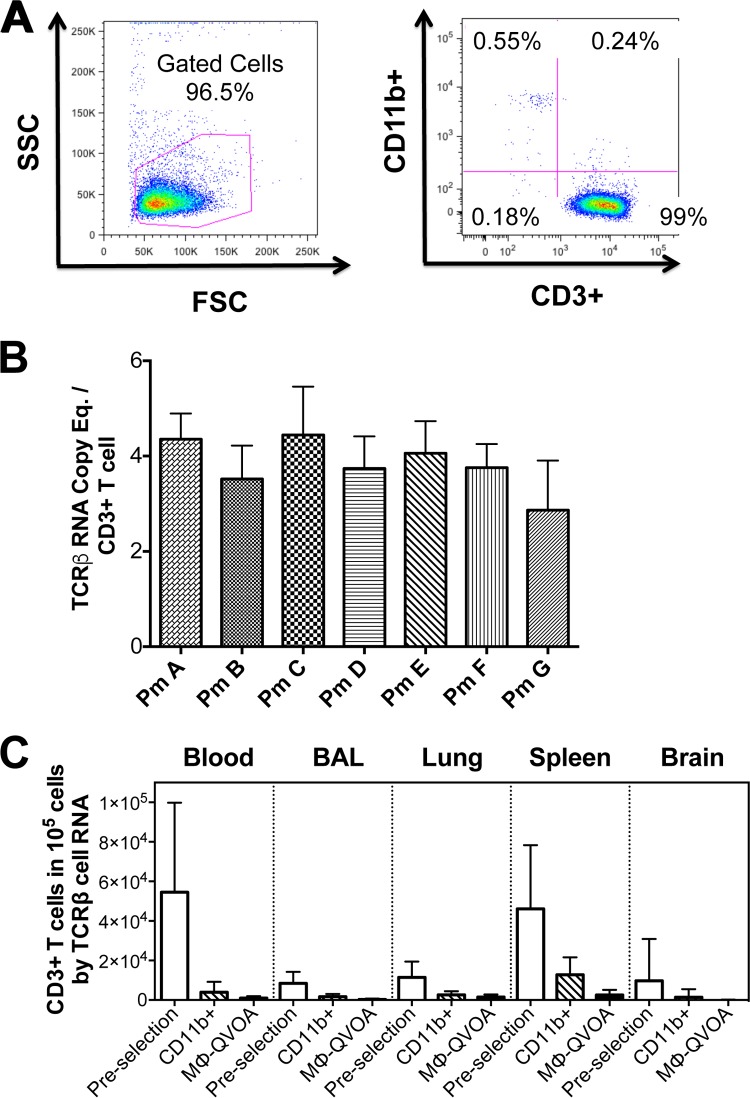
Establishment of the TCRβ RNA assay. CD3^+^ lymphocytes from PBMCs from seven uninfected macaques were purified using an anti-CD3 antibody-positive selection kit. CD3^+^ cells were analyzed by flow cytometry. (A) Representative side scatter/forward scatter dot plots (left) and CD11b/CD3 plots (right) are shown. (B) The average number of TCRβ RNA copies per CD3^+^ T cell was determined by qRT-PCR. Eq., equivalent. (C) The amount of TCRβ RNA in lysates of cells from the indicated compartments from Mϕ-QVOAs was measured by qRT-PCR, and the average number of CD3^+^ T cells per 10^5^ cells was calculated prior to selection, following CD11b isolation, and in CD11b^+^ macrophage control wells from Mϕ-QVOAs. Data are presented as means with standard deviations.

The purity of myeloid cells selected with CD11b-specific beads was assessed by flow cytometry by examining cells that expressed CD3 and CD11b. CD11b^+^ myeloid cells from blood, BAL fluid, lungs, spleen, and brain from 14 SIV-infected macaques were analyzed. Flow cytometry analyses of PBMCs isolated from blood after CD11b^+^ cell selection showed that there were <1% CD3^+^ cells among the selected cells (Fig. 1A). The percentage of CD11b^+^ cells selected from tissues ranged from 94% to 99.1% (Fig. 1B). CD11b^+^ cells adhered to the culture plates, and any residual CD3^+^ lymphocytes that remained in the culture supernatant did not proliferate (Fig. 2C).

To evaluate the presence of contaminating CD4^+^ T cells in the macrophage cultures, CD11b^+^ macrophage control wells without CEMx174 cells were analyzed at 12 days postseeding, the cells were lysed, and TCRβ RNA was quantitated by qRT-PCR. TCRβ RNA is present in macaque CD3^+^ T cells at an average of 3.8 ± 0.8 copies per cell, as it was determined by qRT-PCR (Fig. 2B).

The number of CD3^+^ T cells was quantified prior to CD11b^+^ cell selection, after CD11b^+^ selection, and at the end of the Mϕ-QVOAs in the control wells without CEMx174 cells (Fig. 2C). On average, at the end of the assay less than 0.9% CD3^+^ T cells remained among the monocyte-derived macrophages, 0.3% and 1.5% remained among the BAL fluid and lung macrophages, respectively, 2.5% remained among the splenic macrophages, and 0.06% remained among the brain macrophages. Based on the frequency of infection of CD4^+^ T cells quantitated by the standard QVOA (see Table 3) and the CD4^+^ T cell percentages in the blood and spleen determined by flow cytometry, we calculated that there was, on average, less than one infected CD4^+^ T cell in any of the Mϕ-QVOAs (Table 2). Therefore, the small number of CD4^+^ T cells that remained in the wells was not sufficient to contribute to the amount of virus quantitated in the Mϕ-QVOAs.

**TABLE 2:** Percentages of infected CD4^+^ T cells among macrophages in blood and spleen Mϕ-QVOAs

Compartment and animal identifier^*a*^	No. of IUPM in CD4^+^ T cells	% CD3^+^ T cells in Mϕ-QVOA by TCRβ RNA level	% CD4^+^ T cells among CD3^+^ cells by flow cytometry	% CD4^+^ T cells in Mϕ-QVOA by TCRβ RNA level	% infected CD4^+^ T cells in Mϕ-QVOA	No. of IUPM in CD11b^+^ macrophages
Blood						
Pm4	8.08	0.17	48	0.08	0.0	369.97
Pm5	40.52	1.27	44	0.56	0.2	139. 38
Pm6	205.84	2.01	32	0.64	1.3	23,116.35
Pm7	71.06	0	34	0	0	2.07
Pm8	1,121.51	0	34	0	0	<0.1^*b*^
Pm9	81.70	2.20	31	0.69	0.6	45.96
Pm12	420	0	64	0	0	8.48
Pm13	205.84	1.58	32	0.50	1.0	16.97
Pm14	1121.51	0.31	54	0.17	1.9	4.62
Spleen						
Pm4	40.52	0.36	17	0.06	0.0	423.93
Pm5	9.14	6.23	23	1.41	0.1	854.58
Pm6	40.52	5.74	6	0.32	0.1	93,280.33
Pm9	205.84	2.02	9	0.18	0.4	23.12

a Pm, pigtail macaque.

b Below the limit of detection.

To ensure that SIV gene expression was active in all the infected macrophages, TNF-α, a potent activator of macrophages and the U1 monocytic cell line (54, 55), was added to all wells along with CEMx174 cells to expand replication-competent viruses. CEMx174 is a T/B cell hybrid line widely used to propagate all strains of SIV, including those used to infect the macaques in this study (47, 56).

**FIG 3:**
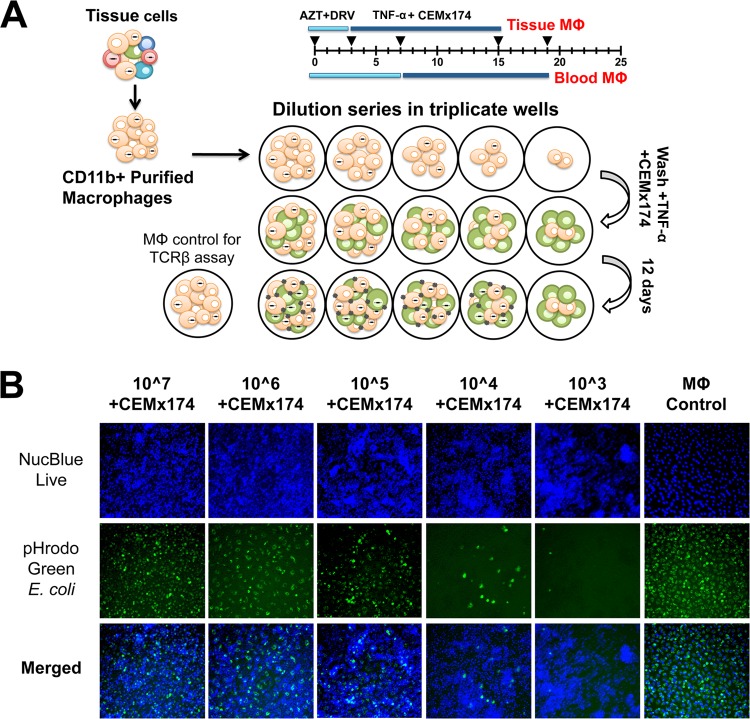
Mϕ-QVOA. Monocytes from blood and tissue macrophages from BAL fluid, lung, spleen, and brain were collected from SIV-infected animals and purified by CD11b-specific bead selection. Macrophages expressing CD11b were plated in serial dilutions in triplicate wells. Cells were cultured with zidovudine (AZT) and darunavir (DRV). Nonadherent cells and the antiretrovirals were removed prior to activation with TNF-α and coculture with CEMx174 cells. (A) Schematic of Mϕ-QVOA. (B) Live fluorescence microscopy of CD11b^+^ monocyte-derived macrophages cocultured with CEMx174 cells stained with NucBlue live nuclear marker (top row, blue) or pHrodo Green E. coli (middle row, green). Merged images are shown in the bottom row. Images were taken on a Nikon Eclipse TE200 microscope. Magnifications, ×10.

Cell supernatants and lysates were isolated separately after 12 days of cocultivation (Fig. 3A). Viral RNA was isolated from cell supernatants from triplicate wells and quantitated individually by qRT-PCR. Wells were considered positive for SIV when RNA levels were higher than 50 copies per 200 μl of supernatant, which was the threshold of detection for the qRT-PCR. The frequency of virus according to the number of infectious units per million (IUPM) was calculated using limiting dilution statistical analyses (32).

To determine the viability of the macrophages cocultured with CEMx174 cells, phagocytosis, a function of viable macrophages, was measured by assessing the number of cells that engulfed pHrodo Green E. coli bioparticles (Fig. 3B). Both CEMx174 cells and macrophages were stained with NucBlue live nuclear stain. The number of cells stained with both markers in each dilution of macrophages reflected the number of viable CD11b^+^ macrophages plated in the well. Despite the numerous CEMx174 cells in the coculture system and the prolonged cell activation, the number of double-labeled macrophages reflected the number originally plated. Furthermore, wells with 10^6^ CD11b^+^ macrophages plated with CEMx174 cells (Fig. 3B, second column) and without CEMx174 cells (Fig. 3B, last column) showed equivalent numbers of double-labeled cells. This suggests that the macrophages remained viable throughout the Mϕ-QVOA, despite the coculture conditions. Finally, Fig. 3B demonstrates that macrophages, unlike lymphocytes, did not expand in culture; therefore, the Mϕ-QVOA provides only a minimum estimate of the size of the reservoir.

### Quantitation of productively infected myeloid cells and CD4^+^ T cells in blood and tissues.

**FIG 4:**
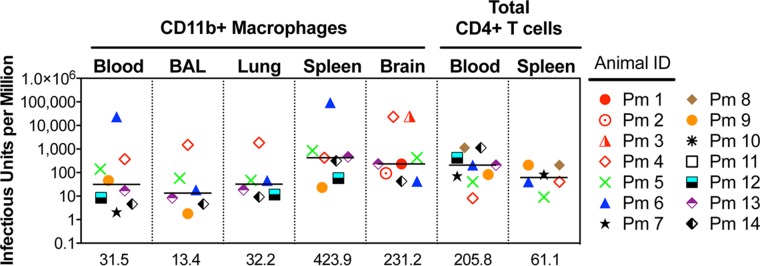
Quantitation of SIV-infected monocytes, macrophages, and CD4^+^ T cells in SIV-infected macaques. Monocytes and macrophages from blood, BAL fluid, lung, spleen, and brain were cocultured in Mϕ-QVOAs. CD4^+^ T cells from the blood and spleen were isolated and plated in a limiting dilution similar to that for the Mϕ-QVOAs. The amount of SIV RNA in the supernatant was measured by qRT-PCR, and the frequency of IUPM was calculated using limiting dilution statistics based on the number of positive wells and the input number of cells. The number of IUPM for macrophages and T cells for each animal assayed is shown. Horizontal black lines, median IUPM values (displayed on the *x* axis); red symbols, animals with severe CNS disease.

**FIG 5:**
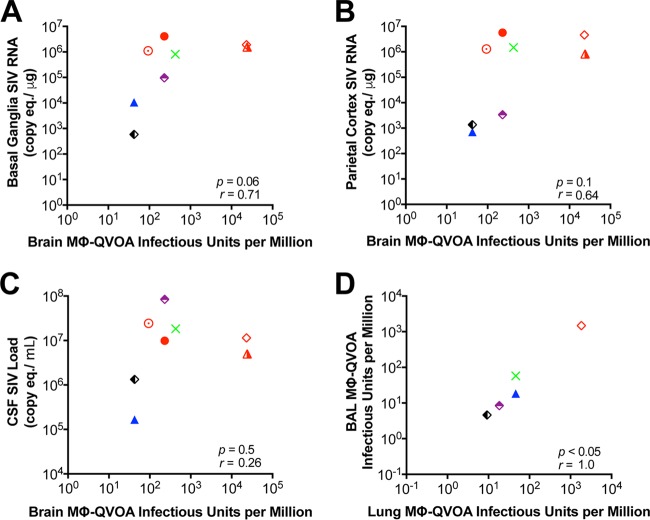
Correlation between the numbers of IUPM obtained by the Mϕ-QVOAs and numbers of SIV RNA copies in tissue. (A to C) Correlation between the numbers of IUPM from brain Mϕ-QVOA and numbers of SIV RNA copies in basal ganglion tissue (A) and parietal cortex tissue (B) and the CSF viral load (C); (D) correlation between the numbers of IUPM in lung macrophages and the numbers of IUPM in BAL fluid macrophages. Each symbol represents a single animal, and symbols in red represent animals with severe CNS disease. Significance was determined by Spearman's rank correlation; a *P* value of <0.05 was considered significant.

The SIV Mϕ-QVOA was used to quantitate the number of productively infected monocytes in blood and macrophages in the BAL fluid, lungs, spleen, and brain of SIV-infected macaques (Fig. 4). The amount of infected macrophages is an estimate of infection on the basis of the assumption that the cells isolated are representative of the population in each tissue. The frequency of productively infected macrophages in each tissue varied among the macaques, with the highest number being found in the spleen (median, 424 IUPM), a secondary lymphoid tissue that contains both CD4^+^ T cells and tissue-resident macrophages. A high frequency of productively infected macrophages was found in macaques with late-stage disease (>84 dpi) as well as those with chronic disease (34 to 36 dpi), suggesting that there is a steady-state level of infected macrophages in spleen or that there is replenishment of infected macrophages throughout infection due to macrophage turnover. In lung, the numbers of productively infected interstitial macrophages were also very similar between the animals with late-stage disease and those with chronic infection; the number of productively infected interstitial macrophages was ∼2.5-fold higher than the numbers of productively infected alveolar macrophages. However, when the numbers of productively infected interstitial and alveolar macrophages within the same macaque were compared, the levels correlated significantly (*r* = 1, *P* < 0.05) (Fig. 5). The interstitial macrophages are derived from blood monocytes, and interestingly, monocyte-derived macrophages from blood had 31.5 IUPM, equivalent to the levels in interstitial macrophages (32.2 IUPM) (Fig. 4).

The majority of the CD11b^+^ cells isolated from brain represent microglia; however, perivascular macrophages, which are monocyte-derived macrophages from blood, also express CD11b. Brain had the widest range of productively infected microglia/macrophages among all the tissues and between macaques. While spleen contained the highest number of infected cells, the brains of animals with mild to severe CNS disease contained the next highest number of infected cells (median, 231 IUPM). The two macaques with the most productively infected cells (Pm3 and Pm4, with 24,000 IUPM) had severe encephalitis and high levels of viral RNA in their brains. The macaques without CNS disease (Pm9 through Pm12) had undetectable numbers of infected microglia/macrophages (Table 3) and little or no detectable viral RNA in the brain (Table 1). The number of productively infected microglia/macrophages trended toward a correlation with the SIV RNA level in the two regions of the brain with the highest levels of viral RNA, the basal ganglia (*r* = 0.71) and parietal cortex (*r =* 0.64) (Fig. 5).

**TABLE 3:** Macrophage and CD4^+^ T cell IUPM in blood and tissues

Animal identifier^*a*^	CNS score	No. of IUPM in the indicated compartment by:						
		Mϕ-QVOA					CD4^+^ QVOA	
		Blood	BAL fluid	Lung	Spleen	Brain	Blood	Spleen
Pm1	Severe					231.16		
Pm2	Severe					93.28		
Pm3	Severe					23,978.95		
Pm4	Severe	369.97	1,481.48	1,838.35	423.93	23,116.35	8.08	40.52
Pm5	Mild	139.38	57.79	46.23	854.58	427.29	40.52	9.14
Pm6	None	23,116.35	18.38	46.07	93,280.33	42.39	205.84	40.52
Pm7	None	2.07					71.06	81.70
Pm8	None	<0.1^*b*^					1,121.51	205.84
Pm9	None	45.96	1.81	<1.0^*b*^	23.12	<0.42^*b*^	81.70	205.84
Pm10	None					<2.31^*b*^		
Pm11	None					<1.05^*b*^		
Pm12	None	8.48	<1.0^*b*^	11.56	57.79	<0.2^*b*^	420	
Pm13	None	16.97	8.48	18.38	462.33	231.16	205.84	
Pm14	None	4.62	4.62	9.19	313.67	42.42	1121.51	

a Pm, pigtail macaque.

b Below the limit of detection.

Productively infected CD4^+^ T cells in the blood had a median of 206 IUPM, a number almost 10-fold higher than the number for infected monocytes in blood (34, 35). However, there was no correlation between the plasma viral load and the frequency of infected macrophages or lymphocytes in a particular tissue or blood. The QVOAs provided a means for comparison of the minimum estimate of the level of productive infection in the two major SIV target cells. Further, they suggested that macrophages are a significant source of virus from tissues during chronic infection.

### Infectivity and sequence analyses of virus from Mϕ-QVOAs.

**FIG 6:**
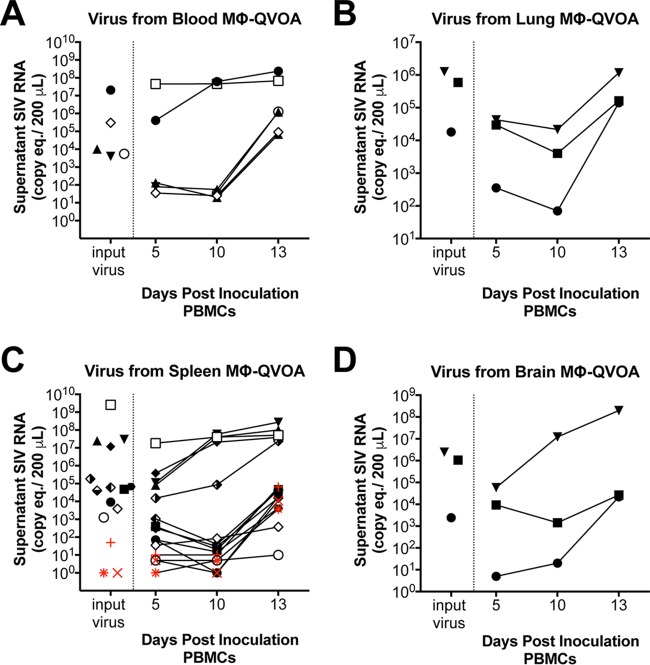
Virus produced in Mϕ-QVOAs is replication competent. Supernatant was collected from the blood Mϕ-QVOA (A), lung Mϕ-QVOA (B), spleen Mϕ-QVOA (C), and brain Mϕ-QVOA (D) for Pm4 (filled symbols) and Pm6 (open symbols) and used to infect freshly isolated PBMCs by spinoculation. The amount of SIV RNA in the supernatant was measured by qRT-PCR prior to infection (input virus) and longitudinally for 13 dpi. SIV RNA levels are presented as the number of copies per 200 μl. Red symbols, wells whose input virus was undetectable.

To confirm that virus produced in the Mϕ-QVOAs was replication competent, virus-containing supernatants from the Mϕ-QVOAs with blood monocyte-derived macrophages, lungs, spleens, and brains from SIV-infected macaques Pm4 and Pm6 were used to infect PBMCs isolated from uninfected macaques. After infection, supernatants from the newly infected PBMCs were analyzed at multiple time points for SIV RNA by qRT-PCR. Viral spread was observed in all wells (Fig. 6B), indicating that all original Mϕ-QVOA supernatants contained replication-competent virus. Interestingly, wells with <50 copies per 200 μl of supernatant in the spleen (Fig. 6C, red symbols) efficiently infected the PBMCs, suggesting that the virus in the Mϕ-QVOAs was infectious even when low levels of SIV RNA were found in the supernatants. This provides evidence that this novel viral outgrowth assay functions like the CD4^+^ T cell QVOA.

**FIG 7:**
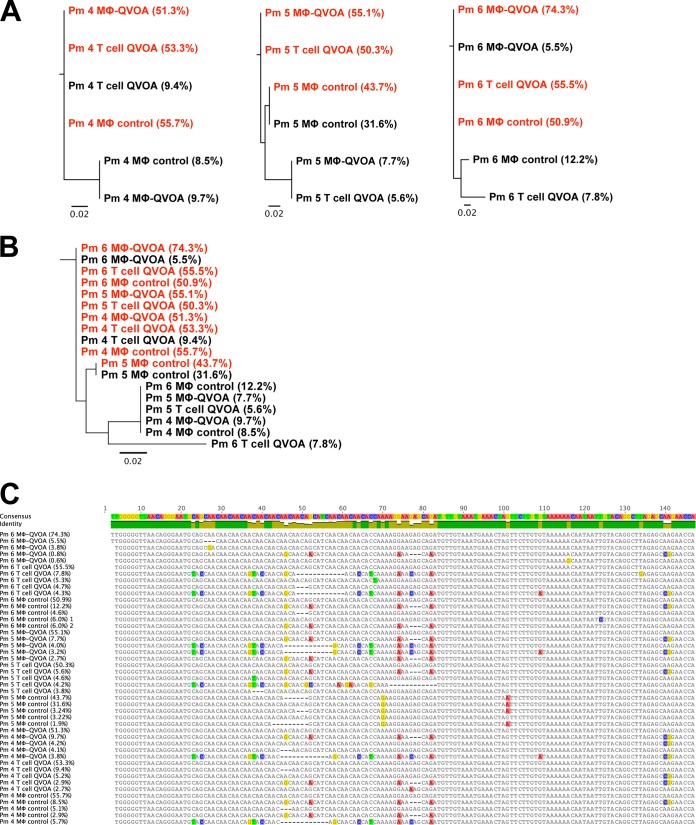
Sequence analyses of virus produced in CD4^+^ T cell and macrophage QVOAs. Supernatant was collected from the spleen Mϕ-QVOA and spleen T cell QVOA for Pm4, Pm5, and Pm6. Viral RNA was isolated, and a two-round nested PCR for SIV *env* was performed. CD11b^+^ macrophage wells without CEMx174 cells from the Mϕ-QVOA were used as controls. The most frequent (red) and the second most frequent (black) sequences are depicted along with the frequency of the viral clone (indicated in parentheses). (A and B) A tree of the nucleotide sequence alignment for each animal (A) and a phylogenetic tree of all clones (B) are shown. (C) Comparison of the nucleotide sequences of the prevailing clones with the consensus sequence, with percentages signifying the frequency of the clones. The scale bar represents the distance between the sequences. Analyses were performed by Geneious (version 8.0) software.

Sequence analysis of the V1 region of SIV *env* was performed with virus isolated from the QVOA wells done with both CD4^+^ T cells and macrophages from the spleens of SIV-infected macaques Pm4, Pm5, and Pm6. The predominant virus found by the Mϕ-QVOA and the CD4^+^ T cell QVOA within the spleen of the same macaque was very similar (Fig. 7A). Furthermore, the viruses produced from CD4^+^ T cells and macrophages from three animals also had very similar sequences (Fig. 7B and C). To exclude the possibility of virus selection from coculture with CEMx174 cells, virus isolated from control CD11b^+^ macrophage wells without CEMx174 cells was also analyzed. Virus from only one macrophage control well had a sequence different from the predominant sequence in the other Mϕ-QVOA wells (Fig. 7). This suggests that coculture with CEMx174 cells did not significantly affect the viruses that replicated and that were detected in the Mϕ-QVOAs. Further, the similarity of the infectious virus isolated from CD4^+^ T cells and macrophages in spleen during chronic infection suggests that the SIVs in spleen during this period are dual tropic, infecting both CD4^+^ T cells and macrophages.

## DISCUSSION

HIV and SIV infection in tissues and *in vitro* has been widely studied. However, the number of macrophages in tissues and monocytes in blood that harbor replication-competent virus has not been quantitated. In this study, we developed an assay to measure the number of blood monocytes and tissue macrophages that contain replication-competent virus. This assay is based on our previous macaque CD4^+^ T cell QVOA. The assay required an understanding that macrophages require culture conditions for adherence and that they do not undergo exponential expansion like CD4^+^ T cells do. The *in vitro* culture of primary tissue macrophages also required different conditions for each tissue that we studied, unlike isolation of CD4^+^ T cells from blood and tissues. In order to select macrophages from tissues, expression of the CD11b antigen was used since it is uniformly expressed on monocytes and macrophages. In addition, to exclude the contribution of CD4^+^ T cells to this assay, we developed an assay for the detection and quantitation of TCRβ RNA.

Using this Mϕ-QVOA, we quantitated the number of macrophages that contained replication-competent virus in the blood, BAL fluid, lung, spleen, and brain of SIV-infected macaques. We demonstrated that macrophages isolated from the blood and from several tissues of SIV-infected macaques harbored replication-competent virus. We also showed that the blood-derived and tissue macrophages used in the Mϕ-QVOA had normal phagocytic function and remained viable throughout the assay, despite prolonged culture and activation with TNF-α. Further, the virus produced by the cells in the Mϕ-QVOA was capable of *de novo* infection of macaque PBMCs. Finally, we characterized the virus isolated from the Mϕ-QVOA by analyzing *env* sequences and virus infectivity in PBMCs. The *env* sequences from viruses isolated from CD4^+^ T cell and macrophage QVOAs were not substantially different. All isolated viruses replicated efficiently in PBMCs, suggesting no selection in macrophages for altered virus tropism.

The number of productively infected macrophages in a given tissue was surprisingly similar from macaque to macaque, whereas the number of productively infected macrophages in different tissues from the same SIV-infected macaque varied widely. The nearly 10-fold difference in the number of productively infected monocytes and the number of productively infected CD4^+^ T cells in blood suggests that monocytes either are less susceptible to SIV infection, have a higher turnover, or harbor more viral genomes that are not replication competent. The highest number of infected macrophages (424 IUPM) was measured in spleen, demonstrating that splenic macrophages are highly susceptible to SIV infection and harbor high levels of productive genomes. This suggests a role for tissue microenvironments in mediating virus infection of macrophages (57, 58). The populations of macrophages that reside in each tissue may be differentially susceptible to SIV/HIV infection on the basis of the cytokine profiles of the organs (59–61).

It has recently been demonstrated that tissues contain two phenotypically different macrophage populations that are derived from either resident (i.e., fetus-derived) macrophages or monocyte-derived macrophages that enter tissues from the bloodstream (62, 63). There are many indications that both types of macrophages harbor persistent virus after suppression of the infection with ART. For example, some HIV-infected patients have a completely controlled plasma viral load yet have detectable virus in the CSF, and some of these patients have accompanying CNS symptoms (64, 65). HIV and SIV infection in brain is predominantly in resident microglia and perivascular macrophages (66–68). In addition, lung inflammation is ongoing in some patients on ART in which HIV infection is suppressed, in part due to infected tissue macrophages (69, 70). ART suppression of virus replication at all stages of disease likely leads to the persistence of infected myeloid cells in tissues. The Mϕ-QVOA that we have developed can be used for human monocytes and macrophages, and the TCRβ RNA qRT-PCR assay can detect transcripts from both human and macaque CD3^+^ T cells. The Mϕ-QVOA will be important for measuring the number of myeloid cells in the tissues of SIV-infected macaques and HIV-infected individuals on ART in which infection is suppressed.

We have previously reported a significant correlation between CNS pathology and an elevated CSF viral load but not an elevated plasma viral load (38); this has also been reported in HIV-infected individuals with CNS encephalitis prior to ART (71). Quantitation of productively infected macrophages in the brain using the Mϕ-QVOA strongly supports the hypothesis that CD11b^+^ microglia/macrophages in the brain are the major contributors to CNS infection since a trend toward a correlation between the number of infected macrophages in both the basal ganglia and parietal cortex of the brain with viral RNA levels but not the plasma viral load was found. It is important to note that even in a model with a high prevalence of CNS pathology similar to that in human disease, not all of the macaques in this study developed severe CNS disease. However, those that did develop mild-severe CNS disease had the highest frequency of infected macrophages in the brain. This study suggests that the frequency of infection of macrophages in the brain is directly correlated with and leads to CNS pathology.

In this study, both blood and tissue from untreated SIV-infected macaques were analyzed because the numbers of productively SIV-infected myeloid cells and CD4^+^ T cells in tissues during infection have not previously been measured. Our results establish that productively infected tissue macrophages can be quantitated, that the virus produced is infectious, and that there is no TCRβ RNA detectable in the infected macrophages. A recent report concluded that tissue macrophages in SIV-infected macaques contained SIV DNA from phagocytosis of CD4^+^ T cells and that tissue macrophages were not a major source of virus *in vivo* (72). In sharp contrast, we developed novel optimized methods for the isolation and evaluation of tissue macrophages, to demonstrate clearly that SIV-infected tissue macrophages produce abundant replication-competent virus. Of note, these novel assays provide a minimum estimate of the amount of productively infected CD4^+^ T cells and myeloid cells in SIV-infected tissues *in vivo*. It is possible that phagocytized CD4^+^ T cells infect macrophages *in vivo*, as it has been demonstrated *in vitro* (73), although a recent study using mouse models has indicated that macrophages can sustain replication *in vivo* independently of T cells (74). Using our techniques, it will now be possible to quantify the number of latently infected CD4^+^ T cells and persistently infected myeloid cells that harbor replication-competent virus in SIV-infected macaques in which infection is suppressed by ART to advance our understanding of HIV latency.
